# Supplementary material for: Are carbon emissions trading and green financial instruments synergistic? -Comprehensive quantitative research based on content analysis
Source: PLoS One. 2024 Mar 7;19(3):e0298601. doi: 10.1371/journal.pone.0298601 (PMC10919601; doi:10.1371/journal.pone.0298601)
Supplement: S1 Data — (ZIP) [file pone.0298601.s001.zip › Data/地级市Z_F工作报告.zip!/║┌┴·╜¡/║╫╕┌╩╨/└┤╫╘╣1⁄2╓┌║┼í╛┬φ┐╦╩2╛▌═°í┐_2003.docx]

各位代表：

　我代表市政府向大会作工作报告，请予审议，并请市政协各位委员提出意见。

　　一、过去五年工作的回顾

　自1997年到现在的五年，全市人民以党的十五大精神为指针，以加快经济发展为中心，大力深化经济体制改革，不断扩大对外开放，积极推进城市转型，努力化解体制性、结构性难题，开创了我市经济发展、社会进步、安定团结的新局面。

　　国民经济快速健康发展。国内生产总值年均增长7.2%，是改革开放以来发展较快的时期，特别是从2001年开始，整体经济增速加快，结束了低速运行的被动局面。财政收入五年平均增长9.8%，比国内生产总值的增幅高2.6个百分点。固定资产投资累计完成85.6亿元，比上一个五年增长58.5%。工业经济在改革调整中实现了新发展，特别是近两年增速加快、效益提高，工业增加值年均增长7％。农业和农村经济快速发展，绿色农业开始起步，产业化经营步伐加快。第三产业发展迅猛，增加值年均增长8%，是三次产业中发展最快的产业。

　　各项改革不断深化。企业改革取得突破性进展，在全省率先推进了地方中小企业改革，产改率达91.7%。农村顺利完成了土地二轮承包和税费改革。大力扶持了非国有经济发展，非国有经济占国民经济的比重由1997年的39.6%增长到51%。建立并完善了养老、失业、医保、城市低保等社会保障体系。积极稳妥地实施了政府机构改革，进一步转变了政府职能。

　　经济结构进一步优化。主导产业发育成熟。在煤炭工业恢复性增长的同时，替代产业不断壮大，电力、化工、食品等产业发展步伐加快，在工业增加值中的比重达到25.1%，对工业乃至整体经济的支撑作用日益突出。农业结构进一步调整，高产、高效、优质农作物比重进一步提高；畜牧养殖业得到较快发展，在农业增加值中的比重达到24.1%；绿色农业稳步推进，绿色食品种植比重达到23.5%。农业产业化发展势头良好，米业、乳业、薯业、菌业四个新兴产业粗具规模。第三产业迅速壮大，房地产业、现代流通业、旅游业、社区服务业等新兴产业全面发展，经济活力日益增强。到2002年，三次产业的比例由1997年的32.6：36.2：31.2调整到24.8：41.0：34.2。

　　对外开放水平显著提高。过去的五年，是我市对外开放最为活跃的时期。对俄开放取得历史性突破。口岸过货量五年增长了18.5倍，跃居全省第六位；口岸功能趋于完善，年装卸能力达到43万吨；对俄经济技术合作发展迅猛，合作领域不断拓展，合作水平进一步提高，合作项目达23个。招商引资成果丰硕。五年累计招商引资89.2亿元，有力支撑了整体经济的快速发展。

　　区域经济发展势头强劲。鹤矿集团实现恢复性增长，2002年首次实现补后不亏。电力工业异军突起，鹤电公司在稳步发展的同时，二期工程取得重大进展。宝泉岭农管局依托资源优势成功运作大项目，成为我省农垦系统投资的重点。鹤北林业局积极推进赴俄森林采伐，为我市发展林木加工业创造了条件。铁路、电业、通信等中省直单位在我市经济发展中发挥了不可替代的作用。

城乡建设取得历史性突破。累计完成交通、通讯、水利、城市基础设施等投资24.7亿元，比前五年增长122%。松花江防洪、黑龙江中心护岸等重点工程进展顺利。鹤佳公路复线、鹤伊公路鹤岗段、鹤名公路相继建成。城乡电网改造按计划推进，完成投资3.2亿元。城市住宅和基础设施建设创造了连续多年建设势头不减、投资规模不减、经济社会效益不减的奇迹。五年新建住宅330多万平方米、康居工程96万平方米，新建、改扩建城市主次干道56条、街巷道路160条，建设了城市供水二期工程、集中供热一期工程，供水、供热能力五年增加一倍，人民群众行路难、吃水难、供热难的问题已经成为历史。新建了一批城市广场、市场商服和公益福利设施，城市功能日益完善，城市形象和知名度大幅提升。

人民生活水平明显提高。城镇居民人均可支配收入年均增长9.1%，农民人均纯收入在灾多灾重的情况下保持了一定增长。城乡居民的居住条件得到极大改善，城市人均住宅建筑面积达到16.8平方米，接近全省平均水平；农村人均住宅建筑面积达到29.1平方米。居民消费结构发生可喜变化，住房、教育、文化、娱乐等方面的支出加大；社会消费品零售总额年均增长5.8%。社会保障能力明显增强，实现了养老保险、失业保险、下岗职工基本生活和城市最低生活保障“四个确保”。城市扶贫解困工作取得明显成效，有9330人脱贫解困。就业和再就业工作得到加强，累计安置就业和再就业7.5万人。

　　各项社会事业全面发展。科技工作长足进步。加强了与科研院所和大专院校的联合，增强了科技创新能力，提高了科技对经济增长的贡献率。教育工作成效显著。基础教育迈上新台阶，提前两年实现“两基”目标，普通高考升学率居于全省上游；高校建设实现了历史性突破，创办了鹤岗师范高等专科学校，结束了我市没有地方高校的历史。卫生、文化、体育事业进一步发展。医疗卫生条件进一步改善，各类传染病发病率明显下降；爱国卫生工作再上新台阶，实现了创建省级卫生城的目标；文化事业进一步繁荣，文化市场管理更加规范有序。广播电视深化了体制改革，改造了传输设施，增加了频道数量，提高了播出质量。群众性体育活动蓬勃开展，竞技体育成果丰硕，在全国和省级运动会上创出佳绩。计划生育工作不断加强，人口自然增长率控制在2.05‰以内。社会治安形势继续好转。各类刑事案件发案率大幅度下降，人民群众的安全感明显增强。此外，民族、宗教、外事、侨务、民政、人防、双拥、环保等各项社会事业都取得了可喜成绩。

　2002年，全市上下积极应对新形势，抢抓新机遇，强力推进“五大工程”，深入实施“五大战略”，经济社会更好、更快地向前发展。国内生产总值实现76.9亿元，比上年增长10.3%，五年来首次实现两位数增长，增幅位居全省第六位。财政收入完成6.9亿元，比上年增长12.9%，总财力创历史最好水平。工业经济速度和效益创历史新高，发展后劲显著增强。工业增加值实现18.7亿元，比上年增长21.2%，其中地方工业实现5.4亿元，比上年增长12.2%。实现工业利税3.9亿元，比上年增长25.8%，其中地方工业实现1.33亿元，比上年增长17%。共实施重点技术改造项目24项，完成投资3.3亿元，扩张了工业总量，推进了结构调整。与此同时，启动了黑龙制瓶一号生产线、二农药、种鸡场、工农洗煤厂、陶粒厂，盘活闲置资产5000多万元。　绿色农业发展步伐加快，农业和农村经济稳步发展。绿色及无公害食品种植面积达到45万亩，比上年增长20%，占总播种面积的23.5%；商品量达到16万吨，比上年增长14%。结构调整步伐加快。扩大了绿色、特色和经济作物种植面积；发展了奶牛、肉牛、山绵羊及特禽养殖，促进了畜牧业“半壁江山”建设。产业化经营开始加快，新兴产业迅速壮大。乳业向规模化、专业化、优质化方向发展；米业的规模优势初步形成；薯业加快了基地标准化建设，扩大了种薯繁育规模；菌业发展成为省内重要的香菇生产基地。城市建设势头旺盛，城市化水平继续提升。2002年是我市城市建设工程量最大、投资额最大的一年，也是近年来建设质量和效果最好的一年。经济适用住房竣工58.86万平方米，比上年略有增长；农村康居工程建设17.4万平方米，为年计划的116%。路桥建设再创佳绩，投资近亿元新建、扩建城市主次干道11条、街巷网化道路99条，改造了胜利桥、团结桥。重点工程建设进展顺利，消防指挥中心、建设大厦、警官培训中心等8个大型工程相继完工。塌陷区治理一期工程进展顺利，完成投资1.4亿元。城市管理水平继续提高。“城市管理年”活动成效显著，八项管理力度不断加大，城市面貌明显改观。对外开放进一步扩大，经济发展的外向水平有新提高。对外贸易快速增长。完成进出口总额364.8万美元，比上年增长121.3%，增幅位居全省前列。对俄经济技术合作向纵深发展。过境种植、养殖、森林采伐、医疗器械生产等合作项目运行良好，实现了多层次、宽领域、跨越式发展。口岸运行又有新突破。口岸过客11847人次，比上年增长25.7%；口岸过货13.7万吨，比上年增长近一倍。招商引资成果喜人。引进项目261项，到位资金达到27.8亿元，比上年增长28.7%。个体私营经济发展迅猛，牵动作用进一步增强。个体私营企业达到2.4万户，其中三型企业达到439户，新增39户。实现工业增加值2.4亿元，比上年增长20%；实现社会消费品零售总额18.6亿元，比上年增长18%；上缴税金9526万元，比上年增长14.8%。

社会事业全面进步，人民生活进一步改善。城镇居民人均可支配收入达到4813元，比上年增长19.7%。农民人均纯收入因灾有所下降，达到2010元；社会消费品零售总额达到24.8亿元，比上年增长8.2%。社会保障得到加强，实现了“四个确保”，保障了社会弱势群体的基本生活，维护了社会稳定。科技、教育、卫生、文化、体育等各项社会事业都取得了新的成就。

　总之，过去的五年，是全市人民在市委的领导下攻坚克难、锐意进取的五年，是整体经济在改革调整中快速发展、再创辉煌的五年，是各项改革不断深化、对外开放取得重大突破的五年，是政通人和、安定团结、社会全面进步的五年，是人民群众生活水平显著提高、得到实惠最多的五年。这些成绩的取得是全市人民解放思想，与时俱进，开拓创新，拼搏实干的结果，是区域内各中省直单位加快发展、争做贡献的结果，是各级人大依法监督和关心支持，各级政协民主监督、参政议政的结果。在此，我代表市政府向全市工人、农民、知识分子和各阶层建设者，向中省直单位和大力支持、积极参与我市经济建设的社会各界人士和朋友表示衷心的感谢！

　纵观五年来的经济社会发展，我们取得了显著的成绩，但还存在亟待解决的问题和困难。主要是整体经济实力相对较弱，综合市力不强；经济结构需要进一步调整，煤炭工业仍占有较大的比重，高新技术产业、现代服务业所占的比重较低；财政压力仍然很大，收支矛盾还很突出；外向型经济基础薄弱，对经济发展的拉动力不强；农民收入增长缓慢，下岗职工多，就业压力大，城乡弱势群体生活困难的问题比较突出；安全生产形势不够稳定，整顿和规范市场经济秩序还需加大力度，经济发展环境有待进一步改善。这些问题影响和制约着我市的经济社会发展，我们要在今后的工作中认真加以解决。

　各位代表，回顾五年来所取得的可喜成绩，我们深切体会到，要推动我市经济社会快速健康发展，必须高举邓小平理论伟大旗帜，认真实践“三个代表”重要思想，全面贯彻党中央、国务院的各项方针政策，认真落实省委、省政府的战略部署，务实求实，开拓创新。第一，必须坚持以加快发展为中心。坚持发展是硬道理，聚精会神搞建设，一心一意谋发展，坚持加快发展不动摇，坚持用发展的办法来解决前进中的问题，努力保持较快的发展速度。第二，必须坚持从市情出发科学谋划发展思路。坚持以市委的总体要求为指导，立足于基本市情谋划经济发展思路，从制约经济发展的主要矛盾入手确定工作重点，从经济发展的现实需要出发明确努力方向，实事求是地组织和指导经济工作。第三，必须坚持把工业放在全市经济发展的首位。坚持把工业放在整体经济发展的首要战略位置，牢固树立“大工业”的思想，通过项目建设、区域联动实现工业总量的扩张、结构的优化、发展后劲的增强，以工业经济的快速增长支撑和保证整体经济的持续、快速、健康发展。第四，必须坚持以项目开发牵动整体经济发展。坚持以项目为载体，围绕优势资源开发项目，围绕提高城市化水平建设项目，围绕对俄开放运作项目，加速推进项目牵动战略，大上快上市场前景广、牵动作用大、经济效益好的项目，以此牵动整体经济发展。第五，必须坚持不断扩大对外开放。坚持立足资源、地缘和政策优势，以北开促南联，把外向求进作为整体经济发展的动力支撑，借助外力求发展，走“无外不快、无外不富、无外不强”的经济发展之路。第六，必须坚持求真务实、艰苦奋斗的作风。坚持以优良的作风推动各项工作，大力弘扬求真务实、脚踏实地、埋头苦干的工作作风，把反映实情、多办实事、讲求实效作为政府最基本的原则，深入实际解决问题；始终保持和发扬艰苦奋斗的光荣传统，以奋发有为的精神状态，攻坚克难，艰苦创业，发展一方经济，致富一方百姓。

　　二、今后五年的工作

今后五年是我市经济社会发展的关键时期，既要全面完成“十五”计划和“五大工程”各项任务，又要为全面建设小康社会奠定坚实基础。因此，政府工作既要顺应时代发展的要求，又要符合实际发展的需要。党的十六大开启了全面建设小康社会的新征程，为我们指明了前进的方向，明确提出了要对东北老工业基地、资源型城市、粮食主产区的发展给予政策支持，为我市加快发展提供了新的机遇。我们要紧紧抓住这一战略机遇期，作好国家政策与我市实际相结合的文章，努力把我市建成国家和我省重要的能源基地、煤化工基地和绿色食品基地，建成联结内地、面向俄罗斯和东北亚的开放前沿，建成以绿色农业、山产品开发、特色旅游为重点的生态经济强市，推动我市走向生产发展、生活富裕、生态良好的健康、文明的发展道路。面对新的形势和任务，今后五年政府工作的总体要求是：以邓小平理论和“三个代表”重要思想为指导，全面贯彻党的十六大精神，深入落实省九次党代会部署，进一步解放思想，开拓进取，围绕增强整体实力和强化财源建设，继续推进“五大工程”，实施“五大战略”，依托项目建设实现经济总量的跨越式扩张，依托结构调整实现地方财力的跨跃式增长，依托整体经济的发展实现人民生活水平的跨跃式提高，依托物质文明、政治文明、精神文明建设实现各项社会事业的跨跃式进步，在建设边疆名城、实现富民强市、全面建设小康社会的新征程中迈出更加坚实的一步。

　　今后五年，我市经济社会发展的主要目标是：

　　——经济发展实现新的跨越。整体实力和综合市力显著提升，经济发展质量大幅度提高，经济结构趋于合理，发展后劲明显增强。工业经济形成支柱企业群体崛起、主导产业发育成熟、区域合力优势明显的新格局，在整体经济中的主导地位更加突出；农业产业化步伐加快，绿色农业迅速壮大，非农产业快速发展，农业综合效益显著提高，农业的基础地位更加牢固；旅游业、现代流通业、现代服务业迅速发展，成为新兴支柱产业。在此基础上，国内生产总值年均增长9%以上。

　　——可持续发展能力大幅度增强。接续和替代产业发展迅速，优势资源得到合理、有效开发；生态环境进一步改善，人口、资源、环境与经济建设协调发展，良性互动；经济增长建立在自然资源深度利用、生态环境有效保护、经济要素合理配置的基础之上。

　　——人民生活向小康目标迈进。城镇居民人均可支配收入和农民人均纯收入有较大幅度增加，用于住房、教育、文化、娱乐、通讯等方面的支出进一步增长；城市和农村人均住宅建筑面积超过全省平均水平；人民群众的生活空间基本优良，建成区域性中心城市，都市化水平大幅度提高，跻身全国卫生城市和文明城市行列，人民群众得到更多的实惠。

　　——社会事业实现新的发展。数字化城市粗具规模，经济和社会信息化建设全面推进，电子政务、企业信息化、电子商务等信息化系统趋于成熟，社会的信息化水平不断提高，科技对经济发展的先导作用明显增强，科技进步贡献率显著提高。教育改革不断深化，素质教育得到加强；各类教育协调发展，教育、教学质量全面提高；地方高校建设迈上新台阶。人民群众的文化生活进一步丰富，精神风貌更加健康向上。医疗服务水平进一步提高，社会保健体系趋于完善。加强体育工作，不断提高全市人民的身体素质。社会保障体系更加完善，保障功能基本健全。与此同时，民族宗教、外事侨务、公安司法、边防安全、双拥人武、计划生育等各项社会事业全面发展。

　到2005年，全面完成或超额完成“十五”计划和“五大工程”的各项任务，总体经济实力大为增强，经济结构进一步优化，地方财力跨越式增加，人民生活水平大幅度提高，基本消除贫困。国内生产总值突破100亿元，财政收入力争突破10亿元，“十五”期间固定资产投资累计突破100亿元，招商引资累计超过100亿元，城镇居民人均可支配收入、农民人均纯收入分别达到6800元、3500元。

　到2007年本届政府届满时，整体经济实现跨越式发展，国内生产总值达到126亿元，比2000年翻一番，提前三年实现翻番目标；财政实力显著增强，财政收入突破12亿元，比2000年翻一番；固定资产投资五年累计突破100亿元，招商引资五年累计超过130亿元。人民生活水平进一步提高，城镇居民人均可支配收入和农民人均纯收入达到全省平均水平，弱势群体得到充分的社会保障。经过五年的努力，综合市力跻身全省中上游水平，人民生活更加富裕、生活质量更加优良，整个社会更加繁荣、文明、进步，成为比较发达的边疆城市，实现全面建设小康社会的第一步战略目标。

　为了实现上述目标，今后五年要重点抓好五个方面工作：

　　——突出抓好项目建设。大力实施项目牵动战略，抓好项目开发和建设。依托农牧、森林、煤炭等优势资源，立足现有的产业基础，瞄准现代先进技术，开发建设能够牵动整体经济跨越式发展的大项目、好项目。重点推进鹤电公司二期工程、煤层气工业化、农副产品加工、林木深加工、鹤矿集团鸟山矿开发、塌陷区综合治理等项目的开发和建设。

　　——全方位扩大对外开放。“南联、北开”并重，把扩大对外开放作为整体经济发展的战略重点。加强与国内先进发达地区的经济技术合作，引进大企业、大集团。提高对俄经贸合作的层次和水平，提升我市在全省对俄开放中的地位和作用。

　　——努力提高城市化水平。打造新鹤岗，建设大鹤岗。加快老城区改造，积极辟建新城区，扩大建成区面积。大力推进城市基础设施建设，现有建成区内的道路桥梁全部改造完成，通乡公路全部达标，网化公路达到高等级标准，加快城市和农村电网改造，供热、供水等基础设施满足经济社会发展的需要。加快小城镇建设，推进农村城镇化进程。

　　——加快改革创新步伐。深化国有资产管理体制改革、政府机构改革、事业单位改革，进一步推进体制创新、制度创新、机制创新，为经济发展和社会进步注入新的动力。

　　——优化经济发展环境。努力规范政府行为，顺应市场经济和招商引资的需要。加强精神文明建设，形成优良的诚信环境、政策环境、法制环境、服务环境，把我市建成万商云集的投资热土。

　　各位代表，2003年是完成新一届政府五年任务的起步之年，也是实现“十五”计划的关键一年，做好今年的各项工作，对于全面建设小康社会具有特别重要的意义。今年全市经济发展的主要预期目标是：国内生产总值增长9%，财政收入增长9—10%(可比口径)，固定资产投资增长12%，社会消费品零售总额增长8.5%，进出口总额增长15%，城镇居民人均可支配收入增长8%，农民人均纯收入增长44%。

　　今年的主要工作任务是：

　　(一)强化工业经济的主导地位，加快工业发展步伐

坚持“工业富市”的战略思想不动摇，把工业放在更加突出的位置。提高项目开发和建设的层次、规模、水平，不断优化产业结构；强化工业经济的运行监控，不断改善运行质量；进一步盘活存量资产，培育发展主导产业，提高工业经济的整体实力。今年工业增加值和利税要分别增长10％。 继续实施项目牵动战略。着力抓好16个重点项目的建设，哈啤（鹤岗）公司年产10万吨啤酒生产线扩产改造、华欣纸业木质素、鹤矿集团安全技术改造等7个续建项目要确保如期完成，华中集团水循环暖床器半自动化生产线改造、鹤嘉腐植酸公司年产5000吨水稻增产素改造等9个新建项目要确保如期开工。

　突出抓好工业经济运行。对产品有市场、发展有潜力、能够快速增长的企业，通过政策倾斜、重点扶持等措施，使其扩大生产规模，提高经济效益，增强拉动能力。对产品市场稳定、生产运行平稳的企业，通过抓市场、抓营销、抓管理、抓服务等措施，促进企业稳定发展。对困难较大、运行艰难的企业，通过政策引导、协调服务、一企一策等措施，促进企业增强活力，重现生机。全力支持鹤矿集团、鹤岗发电公司等中省直企业深化改革，扩大能力，提高效益，加快发展。

　做好关停企业的启动工作。对现有的关停企业重新梳理，分类排队，采取切实有效的措施，进一步加快启动步伐。今年要完成黑龙制瓶大线、大成酒业等4户停产企业的启动工作。

　加快工业园区建设。把建设工业园区作为培育发展主导产业和骨干企业群体，增强工业发展后劲，促进整体经济快速发展的有效载体。要在完善规划的基础上，加快基础设施建设，使园区具有较强的项目承载能力；要制定优惠政策，努力健全服务体系，营造“洼地效应”，吸引更多的企业入驻园区，尽快形成规模。

　　(二)坚持绿色农业发展之路，增加农业综合效益

 围绕培育、壮大优势主导产业，依靠科技进步、调整结构、区域联合、项目拉动、产业化经营，走绿色农业发展之路。农业增加值要达到7亿元，比上年增长15％，实现一年受灾一年恢复。深入实施“绿色农业工程”。进一步扩大绿色及无公害食品种植基地规模，确保突破60万亩，商品量达到20万吨，分别增长33％和25％，总产值达到2亿元。充分发挥绿色食品品牌的辐射带动作用，大力开拓市场，增强市场竞争力。建立和完善龙头企业与基地农户的利益连接关系，在龙头企业的带动下，实现绿色食品种植的快速发展。

 进一步调整和优化农业经济结构。在种植业调整上，主要方向是“两减、三增”，即适度减少水稻、玉米面积，增加大豆、经济作物、饲草饲料面积，通过结构调整促进粮经饲三元种植结构的优化。在产业调整上，将畜牧业调大、升位，加工业调强、调优。畜牧业依托三大乳业集团和宝局200万头生猪加工项目，加快以“两牛一猪”为重点的畜牧业“半壁江山”建设，扩张总量，加速升位。加工业要着力培育、壮大一批规模较大、牵动作用强的龙头企业，为绿色农业工程的推进和农业产业化经营提供保障。加快农业产业化进程。继续推进四个新兴产业加快发展，大力实施“两个振兴计划”，依托宝局的大豆加工、乳制品、生猪加工等大项目，把米业、乳业、豆业、猪业提升为能够拉动全市经济发展的重点产业。米业发展要调整品种，加快基地建设，整合品牌，提高市场竞争力。乳业发展要在提升奶制品总量和档次、扩大市场份额上有大的突破。加快实施“奶牛振兴计划”，区域内奶牛要达到5.5万头，市属奶牛要达到1万头。豆业发展要加快实施“大豆振兴计划”，落实高油大豆种植面积15万亩，使大豆成为农村经济增长、农民增收的新亮点。猪业发展要依托龙头企业，扩大生猪饲养量，全年出栏20万头。菌业发展要扩大以香菇、杏鲍菇、黑木耳为主导产品的食用菌基地生产规模，形成食用菌产业群。薯业发展要扩大种薯繁育和马铃薯种植面积，种薯繁育要达到1.5万亩，脱毒马铃薯种植要达到5万亩。大力实施科技兴农，推广、普及先进适用技术，搞好示范带、示范区建设，提高中低产田种植水平。重点是辟建1条沿黑龙江流域农业科技示范带，创建20个农业科技示范区，落实10项重点科技项目，推广10项重点技术，使农业科技迅速转化为现实生产力。

 继续抓好项目建设。围绕主导产业、绿色生态农业、农业产业化和农业基础设施大上、快上项目。今年要建设50个农业项目，计划投资2亿元，重点要抓好小鹤立河水库扩建、梧桐河防洪治涝等10个项目的建设。

　　(三)全面扩大对外开放，提高整体经济的外向水平

　继续坚持“外向求进”，努力提高外向型经济的比重和整体经济的开放水平。进出口总额要实现420万美元；实际利用外资完成220万美元，同比增长9.8％；口岸过货13万吨，过客1万人次；招商引资到位资金32亿元，同比增长15％。继续扩大对俄经贸合作。一要提高对俄贸易的层次和水平。大力拓展贸易领域，进一步做好粮食、建材、果菜等大宗商品的出口。努力提高贸易层次，积极运作赴俄家电、电脑产品的组装生产，促进家电、电子元件商品的出口。千方百计扩大规模，以大力发展过境旅游带动民间贸易的增长。二要加强项目运作。重点推进12个对俄经济技术合作项目，力争完成5项，赴俄黄金开采要尽快实施，赴俄房地产开发要进行实质性运作，赴俄生产红砖项目要做好选址、签约等工作，宝泉岭农管局赴俄种植3500公顷土地项目要正式启动，合作生产加工人造大理石项目要正式投产。三要加强主体培育和口岸建设力度。在主体培育上，要积极为具备条件的企业办理外贸经营权，大力引进域外主体到我市安家落户，进一步扩大外经贸主体队伍。在口岸建设上，要不断完善口岸基础设施，尽快解决摆渡船和港口装卸设施更新问题，做好第二港区和名山铁路的论证、设计和相应的准备工作；要加大协调力度，简化查验环节，加快验放速度，落实相关政策，优化口岸环境。要做好黑龙江黄金水道的调研和论证工作，充分发挥江海联运的优势，增强口岸的辐射力和影响力。继续做好黑龙江大桥的筹建工作，千方百计争取国家支持，使大桥筹建工作有重大进展。

 加大招商引资工作力度。一要积极探索行之有效的招商引资方式。继续实施全民招商、专业招商、网上招商、专项招商、参会招商“五路并举”的工作措施，积极探索委托招商等新的有效途径，确保招商引资工作再上新水平。专业招商要以定向招商为重点，依托我市丰富的农林、煤炭等资源优势，吸引大企业、大集团到我市投资兴业。网上招商要力争与上海、哈尔滨等城市实现链接，做到信息互动、资源共享。参会招商要认真抓好会展签约项目的跟踪落实，提高合同履约率、资金到位率和项目成功率。二要增强招商引资的实效性。充分发挥资源优势、地缘优势和政策优势，重点推进生产加工型、科技型、高附加值项目的引进，提高招商引资的层次、水平和实效性。三要创建优良的招商引资环境。进一步改善投资环境，着重解决既能招得来、又能留得住的问题。完善联合办公大厅的功能，简化办事程序、提供全方位服务，为外来投资者经商办企业创造优良环境。

 积极开发和利用两种市场、两种资源。既要大力开拓国际市场，又要积极利用外资。在开拓国际市场上，要在沿海发达城市建立我市企业和产品的推介窗口，搭建中外客商和我市产品直接见面的平台。重点扶持、引导斯达水泵、众诚玛钢、鹤嘉腐植酸、华盛木业、华莹玻璃等外向型企业和绿色食品、药品等优势产品扩大出口，提高出口创汇能力。在利用外资上，要突出煤化工、煤层气的开发利用和绿色食品加工，重点做好煤矸石发电、煤层气利用和粮食、蔬菜、山产品深加工项目的利用外资工作。

　(四)大力推进城市化进程，提高对经济社会发展的承载能力

　继续以“518”工程为载体，加大城市建设和管理力度，完善城市设施，强化城市功能，改善城市环境，努力实现城市建设高速度、城市管理新水平、城市面貌大变化。

　城市路桥改造要全部完成。投资9000万元，新建、扩建胜利路、西解放路、工厂路、新兴路等13条主次干道、40条街巷网化道路，拓宽改造奋斗桥和支农桥。改造完成零公里至南风井公路。经过努力，全市建成区内的主次干道和桥梁全部改造完毕，实现主次干道畅通无阻、街巷道路成环成网、桥梁改造美观新颖、城市交通四通八达，真正形成一个风格现代、功能齐全、高层次、高品位、符合长远需要的城市道路交通体系。

　　继续抓好经济适用住房建设。全年计划开竣工50万平方米，在工农区进行弘景花园、祥和小区、成龙小区的建设，在向阳区加快振兴小区、胜利小区的建设，在东山、南山、兴山、兴安四个区启动中心区改造和住宅小区建设工程。康居工程建设要完成10万平方米，建设6－10个基础设施比较齐全的示范村。全面完成“八大建筑”建设任务。向阳集贸市场、龙华大厦等八大公益福利、商服设施要加快建设速度，按时完成。开工建设振兴广场。

　绿化工程要有新突破。今年城市绿化总的要求是，成片植树，见空栽树，大树进城，自然生态。对新建设的天祥路、工厂路、西解放路等城市主次干道及住宅小区进行重点绿化，进一步开展城市塌陷区、废弃地的绿化，继续实施森林进城。全年要完成专业植树3000株，义务植树30万株，大树进城300棵，塌陷区、废弃地绿化5000亩，使我市在建设生态园林城市上有新的突破。

　亮化工程要上新水平。临街的单位和店铺都要进行高品味的亮化装饰，城市主次干道、主要桥梁的灯饰亮化要加大力度，使楼灯各具特色、路灯流光溢彩、桥灯典雅大方、门灯一片通明，让夜晚的鹤岗更加绚丽多姿。继续深入抓好城市管理。加大烟尘治理力度，采取有力措施，重点对工业锅炉、浴池锅炉及特种行业用气炉加强整治，废除大烟囱20个、居民平房小烟囱3000个。环境卫生要继续保持垃圾日产日清，突出主干道全天候保洁，杜绝尘土扬灰。加强小区管理，从绿化、硬化、美化入手，开展达标准、上等级、创名牌活动，力争多创建省级、市级文明小区。与此同时，努力保持八项管理齐头并进，使城市管理上层次、上档次、上水平，确保城市管理年活动决战决胜，城市面貌有更大的变化。

　(五)加快非国有经济战略升级，增强对整体经济发展的牵动作用

　深入调整所有制结构，推动非国有经济发展速度加快、质量提高、结构优化，增强对整体经济发展的牵动作用。个体私营经济要实现税收1.1亿元，同比增长12％；工业增加值实现2.9亿元，同比增长18％；“三型”企业达到469户，新增30户。强化指导，在改善非国有经济发展环境上要有新突破。继续推进“领导包企”、“警企双百”、“银企对接”、“科企联系”四项重点工作。“三型”及重点私营企业要纳入市级领导包扶范围，加大扶持力度；扩大银企对接范围，缓解个体私营企业发展的资金瓶颈制约；加强科技机构与私营企业的联系，积极推动企业科技进步。进一步完善政策规定，在市场准入、资金融通、信用担保等方面提供更加广阔的发展平台。狠抓政策落实到位，严肃查处“三乱”和干扰企业正常经营的行为，维护企业的合法权益，为非国有经济发展创造良好环境。

　强化引导，在发展“三型”企业、优化经济结构上要有新突破。对原料有来源、产品有市场、科技含量高、增值潜力大的企业，积极帮助其扩大生产规模，加快技术改造，提高产品档次，促其成为“龙头企业”。要以发展“三型企业”为重点，优化产业结构。围绕矿产资源和农业资源，大力发展非煤产业、绿色食品和消费品加工业，向加工型延伸；引导个体私营企业多渠道、多形式引进科技成果，向科技型延伸；支持有条件的企业在境外办企业或开展经贸活动，向外向型延伸。要大力发展社区经济，拓宽非国有经济发展领域。积极引导个体私营企业组建行业协会，培育壮大企业集团和企业联合体，加强企业间的交流与合作，优化资源配置。

　　强化服务，在提高个体私营业者素质上要有新突破。全面启动鹤岗市个体私营经济网站，加强信息服务，开阔私营业者的视野；采取办班培训、专家讲座等方式，有计划地组织私营业者学习现代经营管理、现代科技等知识，提高经营者素质。引导个体私营经济用能人治企，引进现代管理制度和高新技术，增强竞争能力。

　(六)推动社会全面进步，实现经济社会协调发展

　　科技工作要上新水平。加快数字化城市建设速度，启动鹤岗数码城，大力推进企业信息化建设，全面加强中介机构建设；积极开展横向经济技术合作，加快高科技项目的开发和建设，推进产业结构升级。教育工作要有新发展。全面实施素质教育，加快教育教学改革进程，努力提高师生的德育水平、法制水平和创造能力，抓好规范化学校建设，鼓励扶持民办教育，整合职业技术教育，提高全社会再教育率，推进教育现代化。卫生工作要有新成绩。要深化医药卫生体制改革，加快社区卫生服务体系建设，改革卫生监督体制，加强农村卫生和预防保健工作，整顿和规范医疗市场，进一步提高医疗技术和服务水平，保证人民群众健康；爱卫工作要上新台阶，向创建全国卫生城市迈进。文化事业要全面发展。深化文化体制改革，加快文化设施建设，繁荣文艺创作和文艺舞台，广泛开展群众文化活动，丰富人民群众的生活；广播电视工作要努力创办人民群众喜闻乐见、丰富多彩的广播电视节目，要迅速完成有线电视网络的整合工作，扩大网络覆盖面，让更多的人民群众享受到高质量的电视服务。体育工作要以提高全市人民身体素质为目标，开展全民体质测试工作，以中小学生为重点开展全民健身运动，竞技体育要扩大项目范围，全面做好备战省十一届运动会的各项工作，再创新佳绩。要继续做好计划生育工作，人口自然增长率要控制在5‰以内。社会保障要有新成效。加强养老金、失业金的收缴和扩面，确保足额及时发放；做好下岗职工基本生活费向失业保险并轨工作；城市居民最低生活保障努力实现应保尽保；积极向上争取，确保社会保障的资金需要。积极做好就业和再就业工作，认真落实中央和省有关就业和再就业的各项优惠政策，加大培训力度，全年安置2万人再就业，培训1.5万人次。要提高依法治市水平，认真开展“四五”普法教育，创造优良的法制环境。要进一步搞好社会治安综合治理，深入开展严打斗争，严厉打击各种刑事犯罪，为人民群众的生活、工作营造良好的治安环境。安全工作要有新提高。继续深入开展五项专项整治，抓好对重点行业、重点企业的监控，最大程度地消除事故隐患；落实各项防范措施，依法强化监督管理，把安全生产监督纳入法制化轨道，督促企业建立安全生产的自我约束机制和长效机制，提高综合防御能力，从源头上防止重特大事故的发生。此外，民族宗教、外事侨务、民政档案、国家安全、双拥人防等各项社会事业都要取得新进展。

　三、全面加强政府自身建设

　今后五年，我们肩负着全面建设小康社会的历史使命，作为新一届政府，我们必须以十六大精神为指导，以坚持发展的思想、创新的精神和务实的作风为基本工作原则，科学定位角色，加快职能转变，提高驾驭市场经济的能力，树立全新的政府形象，建设一个符合时代特点、顺应时代要求、体现时代精神的新型政府。

　第一，建设学习型政府。建设学习型政府，就是要主动适应“入世”和知识经济的挑战，在各级政府和政府各部门中大兴学习之风，全面提高领导干部和公务员队伍素质，这既是政府自身建设的客观要求，也是创建社会主义政治文明的现实需要。每一名公务员特别是领导干部要不断加强学习，使学习成为经常化、普遍化和制度化的自律行为。在加强学习的过程中，要善于挑战自我、超越自我，不断追求思想上的新境界，把自己造就成创新型、复合型人才。要学习世界上一切优秀文明成果，把最先进、最能反映时代要求和人民利益的思想认识和实践经验，体现在我们的思想、方式、方法和行为中来，不断提高科学判断形势的能力、科学决策的能力、驾驭市场经济的能力、应对复杂局面的能力、依法执政能力和总揽全局的能力，增强凝聚力和号召力。要加快政府管理的民主化、法制化进程，提高政治文明的层次和水平。在管理公共事务中，管理手段、方式方法要不断推陈出新，始终站在时代的前沿，以适应新形势、新任务的需要。

　第二，建设责任型政府。强化政府的责任意识，是人民政府的本质决定的，也是我国加入世贸组织的现实要求。一要强化政治责任。要以对党对人民高度负责的态度和精神，努力发展经济，尽快提高人民生活水平；加快城市基础设施建设，改善人民群众的生活质量；关心人民群众疾苦，注重解决实际问题，特别要关注弱势群体，维护社会稳定。二要明确行政责任。加强对县区级政府的行政监督，加强对政府各部门、各单位的管理，重点是通过明确程序、严明纪律、完善制度，加强对政府组成人员和执纪执法部门的有效监管，防止“权力部门化、部门利益化、利益合法化”现象的发生。三要认清法律责任。各级政府、政府各部门、各单位都是行政法的主体，入世更强化了我们的法律责任，为此必须要大力推行依法行政，通过完善法制建设、强化法律监督、落实行政执法责任制，切实提高政府工作的法制化、规范化水平。要尊重人大的法律地位和人民代表的民主权利，自觉接受人大的法律监督，支持政协发挥协商监督职能；要定期向人大报告工作，向政协通报经济社会发展情况；认真办理人大代表、政协委员的提案、议案，虚心听取人民群众的建议、批评和意见，使人民的意愿在政府工作中得到体现。

　第三，建设服务型政府。以改革审批制度为核心，加快政府职能转变，将应该转移的职能交给市场、企业和社会，能由市场调节的交由市场调节，应由企业处理的交由企业处理，应由中介组织承担的交由社会中介组织承担；以推进电子政务为基础，努力提高行政效率，按省里规定的时间完成设备安装、网络组建、人员培训等工作，尽快实施网上办公；以发展社会中介组织为载体，增强社会自治能力，引导各方加大投入，组建行业协会、商会等各类中介组织，规范现有中介组织的行为，提高中介组织为市场服务、为企业服务、为社会服务的水平，积极推进城市社区建设和农村自治建设，提高城市居民和村民的自治能力；以扩大政务公开为关键，提高政府工作透明度，加强社会监督、舆论监督和群众监督，进一步完善人民群众参与经济社会管理的机制。

 第四，建设效能型政府。要完善管理手段，科学定位角色，从繁杂的具体事务中抽出身来，集中精力解决好关系经济社会发展和人民群众切身利益的重大问题。一要履行好调控者的职责。加强对全市整体经济的宏观调控，完善市场经济体制，确保整体经济快速增长，特别是针对经济结构不合理等深层次矛盾，进一步探索调控手段，加大结构调整力度。二要突出公益者的职责。要致力于社会保障体系的健全和完善，保障人民群众的基本生活；要致力于人民群众生存质量的改善，大力加强城市管理，治理污染，保护环境，增加公益福利设施；要致力于创建优良的经济发展环境，规范行政行为，简化办事程序。三要强调管理者的职责。加强对市场的管理，大力整顿和规范市场经济秩序，严厉打击各类违法犯罪行为，公正调节各种利益主体之间的关系，营造公平良好的社会环境。总之，要形成服务全社会、为经济和社会发展提供保障的政府工作新格局，以卓有成效的政府工作带动全市的经济发展和社会进步。

　第五，建设廉洁型政府。保持清正廉洁，是党和人民对政府的基本要求，政府组成人员特别是领导干部要清正廉洁，为党为公，严于律己，树立形象。坚决贯彻执行中央、省关于廉洁自律的各项规定，加强自身修养，增强拒腐防变的能力。认真落实党风廉政建设责任制，严肃查处各种违纪违法案件，纠正部门和行业不正之风，狠刹奢侈浪费之风。从完善制度入手，从严治政，继续实施政府采购制度，认真实行“收支两条线”管理，规范建筑市场，进一步完善公开招标制度。支持和加强审计监督，充分发挥审计监督在经济建设和廉政建设中的作用。深入开展反腐败斗争，坚持标本兼治，重在治本的方针，从源头上预防和治理腐败，严肃查处大要案，严厉打击和清除腐败分子。

　各位代表，历史已经翻开了崭新的一页，展望新世纪全面建设小康社会、实现富民强市宏伟目标的壮丽前景，我们充满必胜的信心和力量。我们坚信，有十六大精神的正确指引，有市委的坚强领导，有百万人民渴求发展的强大动力，我们万众一心，奋发图强，就一定能够为全市人民创造出更加幸福的生活、更加美好的明天!
